# Explainable multi-modal machine learning for predicting occult pulmonary metastases in differentiated thyroid cancer: a SHAP-based approach prior to radioactive iodine scans

**DOI:** 10.3389/fmedt.2025.1685088

**Published:** 2025-11-28

**Authors:** Yuqi Su, Yuhuang Cai, Shui Jin, Xuemei Ye, Jaesik Jeong, Ye Yuan, Heqing Yi

**Affiliations:** 1 Postgraduate Training Base Alliance of Wenzhou Medical University, ZheJiang Cancer Hospital, Hangzhou, Zhejiang, China; 2 Department of Nuclear Medicine, Zhejiang Cancer Hospital, Hangzhou, Zhejiang, China; 3 Department of Mathematics and Statistics, Chonnam National University, Gwangju, Republic of Korea; 4 School of Mental Health, Wenzhou Medical University, Wenzhou, China

**Keywords:** thyroid cancer, machine learning, prediction model, lung metastases, ^131^I treatment

## Abstract

**Background:**

Patients with differentiated thyroid cancer (DTC) may have occult lung metastases before ^131^iodine (^131^I) treatment. Identifying occult lung metastases before ^131^I treatment is of great clinical value for the correct staging of patients and the establishment of ^131^I treatment plans. Our research is of great significance in establishing statistical models for clinical data using machine learning algorithms to study the prediction of lung metastasis before ^131^I treatment.

**Methods:**

Patients were selected from Zhejiang cancer hospital and data was from two groups of DTC patients treated with ^131^I, where the experimental group consisted of 55 patients who showed no lung metastases on CT but tested positive on ^131^I-whole body scan (^131^I-WBS). The control group included 316 patients who tested negative for metastases across CT, ultrasound, and ^131^I-WBS. Six machine learning algorithms such as Support Vector Machines (SVM), Decision Trees (DT), Random Forests (RF), Logistic Regression (LR), Extreme Gradient Boosting (XGBoost), and K-Nearest Neighbors (KNN) were employed to predict models and AUC, sensitivity, accuracy, precision, specificity, F1 Score were used to compare the performance between each models. Finally, the SHAP algorithm was used to explain the importance rank of the features.

**Results:**

A total of 371 thyroid cancer patients were included in this study, 55 patients with occult lung metastasis and 316 patients in the control group. The data is divided into a training set and a testing set in a 7:3 ratio. Eleven acceptable variables analyzed including gender, age, T stage, N stage, tumor size, degree of invasion, number of lymph node metastases count, Thyroid Stimulating Hormone (TSH), thyroglobulin (Tg), Thyroglobulin antibodies (Tgab), and administrated activity were screened out by multivariate Cox regression. Evaluation indicators of the best model- LR were as following: accuracy (0.91), recall rate (0.64), precision (0.92), F1-s core (0.70), Area Under Curve (AUC) value (0.93), and the Specificity score (0.96).

**Conclusion:**

The logistic model (LR) showed the best performance in predicting occult lung metastases of thyroid cancer patients before ^131^I-WBS. Lymph nodes metastases and throglobulin have the most significant impact on the prediction.

## 1 Introduction

Thyroid cancer is one of the most common malignancies of the endocrine system, with increasing incidence rates worldwide, partly attributed to enhanced detection methods (1, 2). Based on the origin and differentiation of the tumor, thyroid cancer is classified into several types: Papillary Thyroid Carcinoma (PTC), Follicular Thyroid Carcinoma (FTC), Medullary Thyroid Carcinoma (MTC), Poorly differentiated thyroid carcinoma (PDTC), and Anaplastic thyroid cancer (ATC), with PTC being the most prevalence, accounting for approximately 90% of all thyroid cancers (3). PTC and FTC together are referred to as differentiated thyroid carcinoma (DTC). With the widespread use of diagnostic technologies such as high-resolution ultrasound and fine-needle aspiration biopsy, the global incidence of thyroid cancer—particularly small, subclinical papillary carcinomas—has risen substantially over the past two decades. Recent studies suggest that much of this increase reflects overdiagnosis rather than a true rise in clinically significant disease (1). This trend has prompted ongoing debates about the potential harms of overtreatment in indolent cases (4).

Typically, DTC has a favorable prognosis. However, the overall survival rate significantly decreases when distant metastases occur. Approximately 10% of PTC and 25% of FTC patients experience distant metastases (5), and due to high hemodynamics, the lungs are the most common site (6), accounting for 55%–85% of all distant metastatic cases. The occurrence of lung metastases significantly complicates the clinical management and worsens outcomes in DTC patients (5, 7–9). Early diagnosis and active treatment of lung metastases in DTC can lead to a 10-year survival rate as high as 90% (10).

Treatment options for DTC with distant metastatic differentiated thyroid cancer include surgical resection, treatment with radioactive Iodine-131 (^131^I), and Thyroid Stimulating Hormone (TSH) suppression therapy (11). Approximately 26%–60% of patients with distant metastatic DTC progress to being refractory to radioiodine (Radioiodine Refractory, RAIR) (12). Among patients with pulmonary metastases from differentiated thyroid cancer (DTC), those with high radioiodine uptake demonstrated a 10-year overall survival (OS) rate of approximately 64%, while those with low or no uptake had a significantly poorer prognosis, with 10-year OS rates dropping below 10% (13). Postoperative re-staging in patients with DTC is a critical determinant in guiding the selection of appropriate ¹³¹I therapeutic activity. In cases with distant metastases, the administered activity of the initial ¹³¹I therapy plays a pivotal role in influencing patient prognosis. Therefore, accurate assessment of postoperative disease status—particularly the identification and evaluation of pulmonary metastases—is essential for optimizing therapeutic decision-making and improving clinical outcomes (14, 15).

Ultrasound is the preferred imaging modality for screening thyroid nodules and assessing their risk of malignancy (16). However, while it can evaluate the malignant potential of thyroid nodules, it cannot determine whether the nodules are likely to metastasize to the lungs. Computed tomography (CT) plays a crucial role in screening for pulmonary metastases in patients with DTC (17). In some DTC patients with occult pulmonary metastases—micrometastases detectable only by ^131^I-WBS—may be present despite negative chest CT findings (13). Consequently, the presence of pulmonary metastases cannot be definitively identified prior to ^131^I therapy, even in patients with elevated stimulated thyroglobulin (sTg) levels. This lack of reliable indicators complicates the determination of the appropriate administrate ^131^I activity for initial therapy, potentially resulting in suboptimal treatment activity.

The application of machine learning in medical imaging has shown promising results in refining diagnostic accuracies and reducing the reliance on invasive tests. Machine learning models are capable of analyzing complex datasets to identify patterns that may elude conventional analysis, potentially predicting clinical outcomes with high precision (18). For instance, studies have successfully used machine learning models to predict various clinical outcomes, including the likelihood of metastases in cancers (19).

This study seeks to build on these advancements by utilizing a unique cohort of DTC patients treated with ^131^I, focusing particularly on those without initial signs of lung metastases on traditional imaging but later confirmed via ^131^I-whole body scan (^131^I-WBS). Unlike the broader epidemiological approaches typically found in the literature, which often utilize databases such as the Surveillance, Epidemiology, and End Results (SEER) for model training and validation (20), our study employs a detailed clinical dataset that includes additional variables such as TSH levels, Tg, and detailed histopathological classifications.

Utilizing six different machine learning algorithms—Support Vector Machines (SVM), Decision Trees (DT), Random Forests (RF), Logistic Regression (LR), Extreme Gradient Boosting (XGBoost), and K-Nearest Neighbors (KNN)—this research aims to explore the feasibility of predicting lung metastases prior to the use of ^131^I-WBS. Each algorithm offers distinct advantages in handling various aspects of predictive modeling, from handling unbalanced data with RF to capturing non-linear relationships with XGBoost (21).

In summary, this study advances the application of machine learning in the management of DTC by developing a predictive model aimed at identifying occult lung metastasis based on routinely available clinical data. Beyond offering a potentially effective tool for early detection, this research contributes to the broader effort to integrate machine learning into routine clinical workflows. By enabling earlier therapeutic interventions, the proposed approach may help reduce reliance on invasive diagnostic procedures, enhance patient outcomes, and optimize the use of healthcare resources.

In this study, “occult pulmonary metastases” are defined as metastatic lung lesions that are not detectable on pre-therapeutic chest computed tomography (CT) scans, but become evident on post-therapeutic ^131^I-whole Iodine Scans (^131^I-WBS) due to radioiodine uptake. Clinically, these metastases are often referred to as “micrometastases” that escape detection by conventional imaging but demonstrate functional iodine-avid activity. This operational definition is consistent with prior literature on iodine-avid but CT-negative metastatic lesions in DTC patients. Identifying such cases is of high clinical importance, as they influence staging, treatment planning, and prognosis despite being radiologically occult (17). This study directly addresses an important clinical gap — the lack of effective tools for predicting occult lung metastases before ^131^I treatment — by leveraging machine learning to assist in early decision-making and individualized therapeutic planning.

## 2 Materials and methods

### 2.1 Research framework

**Figure 1:**
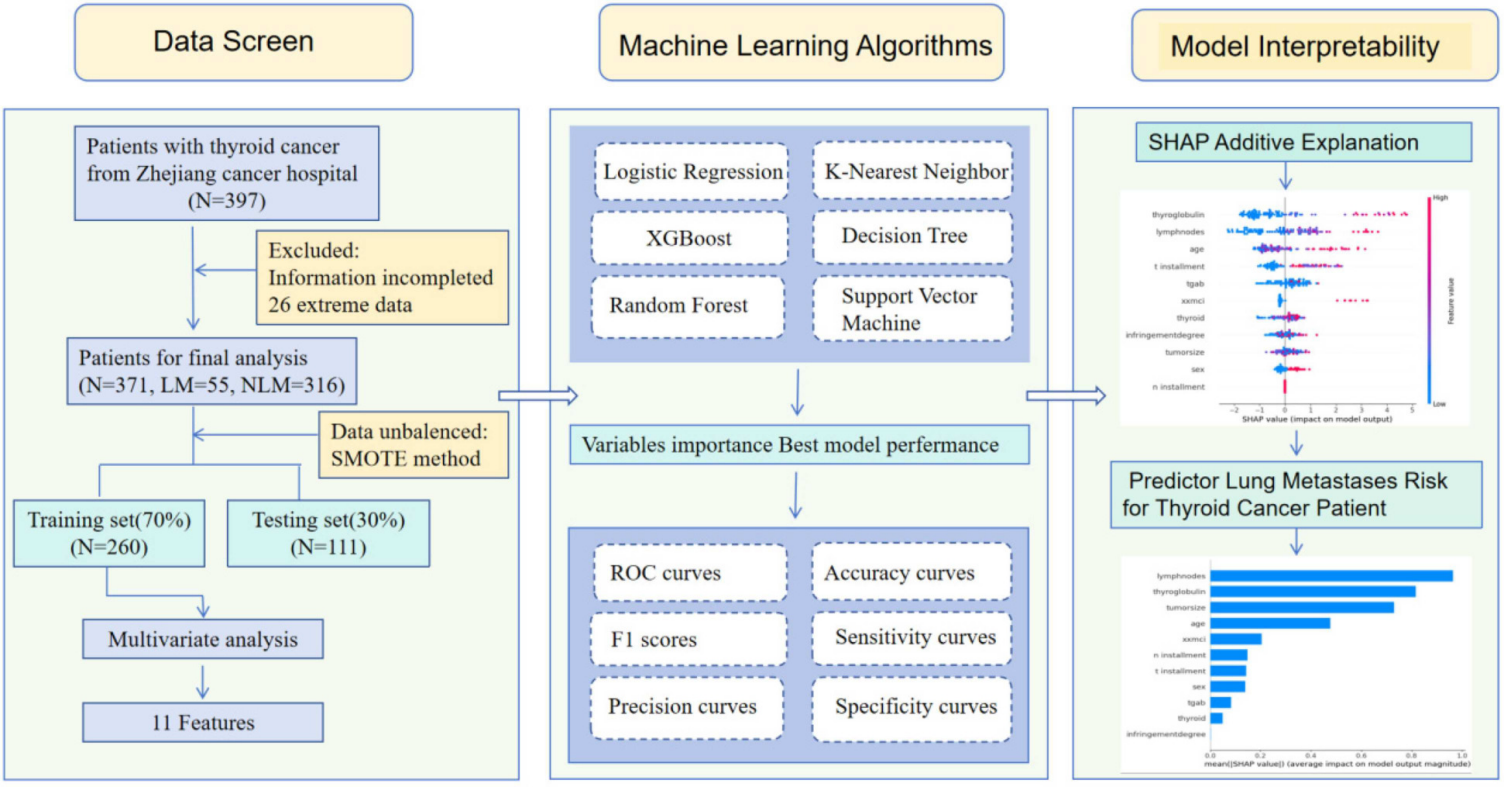
The overall flowchart of the research.

This prediction research utilized information from Zhejiang cancer hospital to construct a binary classifier for predicting Pre-^131^I-WBS positive diagnosis in thyroid cancer. The entire architecture process is illustrated in Figure 1.

### 2.2 Data sampling

In this study, we retrospectively enrolled 371 patients with histologically confirmed differentiated thyroid carcinoma (DTC) who were treated at Zhejiang Cancer Hospital between July 2008 and December 2024. The patients were divided into an experimental group and a control group. The experimental group comprised 55 patients who underwent ^131^I-WBS therapy and presented with no evidence of lung metastases on CT or lymph node metastases count on ultrasound, yet demonstrated positive findings for lung metastases on post-therapeutic ^131^I-WBS. The control group included 316 patients who also received ^131^I therapy but showed no signs of lung or lymph node metastases count on CT, ultrasound, or ^131^I-WBS.

Inclusion Criteria:

1. Histologically confirmed diagnosis of DTC between July 2008 and December 2024;
2. Age ≥ 18 years at the time of diagnosis;
3. No evidence of lung metastases on CT and lymph node metastases count on ultrasound, with ^131^I-WBS indicating lung metastases; or No evidence of metastases on lung CT, lymph node ultrasound, and ^131^I-WBS.

Exclusion Criteria (Clinical Justification):

Patients were excluded from the study if any of the following criteria were met:

1. missing pathological data, including T stage, N stage, or total lymph node count, which are essential for accurate TNM staging and metastatic risk stratification;
2. absence of thyroglobulin (Tg), thyroglobulin antibody (TgAb), or TSH values, which are core biomarkers for post-treatment monitoring and risk assessment in DTC management;
3. missing tumor size or invasion grade, both of which are key variables linked to metastatic potential;
4. non-DTC histologic subtypes such as medullary thyroid carcinoma (MTC), poorly differentiated thyroid carcinoma (PDTC), or anaplastic thyroid carcinoma (ATC) were explicitly excluded through histopathologic review to maintain a homogenous cohort focused on papillary and follicular types;
5. patients with incomplete imaging records or unclear diagnosis status regarding pulmonary metastases were excluded. In addition, although stimulated thyroglobulin (sTg) levels are recognized as more reliable post-ablation predictors, our dataset primarily utilized basal Tg measurements due to institutional standard practices and retrospective limitations. Subgroup analysis by sTg levels was not performed due to lack of uniform stimulation protocols.

### 2.3 Clinical variables selection

A total of 11 variables were collected, including gender, age, N stage, T stage, lymph node metastases count, tumor size, TgAb, degree of invasion, Thyroid Stimulating Hormone, thyroglobulin, activity. All patients included in this study underwent total thyroidectomy as part of their initial treatment. Central neck lymph node dissection was performed routinely, with lateral neck dissection conducted when clinically indicated. The lymph node dissection strategy included both “prophylactic dissection” in patients without radiologically or clinically evident lymph node involvement, and “therapeutic dissection” in cases where metastasis was suspected or confirmed by imaging or fine-needle aspiration (FNA). All surgical procedures were performed according to standard guidelines under the supervision of experienced endocrine surgeons at Zhejiang Cancer Hospital.

### 2.4 Model training and optimization

The dataset from Zhejiang Cancer Hospital was randomly divided into a training set (70%) and a testing set (30%) to minimize the risk of overfitting and ensure robust model validation. Prior to model construction, data preprocessing was performed to normalize and standardize all numerical variables, thereby maintaining consistent feature scaling across inputs and improving model convergence. The preProcess function in the caret package (R, version 4.3.0) was applied to transform each variable into a standardized distribution with a mean of 0 and a standard deviation of 1. Such normalization is essential because differences in data magnitude can significantly influence model optimization and training efficiency.

The selected eleven clinical and biochemical variables were then used as input features to construct six machine learning models: Decision Tree (DT), K-Nearest Neighbor (KNN), Extreme Gradient Boosting (XGBoost), Support Vector Machine (SVM), Random Forest (RF), and Logistic Regression (LR). Each model offers complementary advantages for classification tasks. The SVM model functions as a binary classifier by identifying the optimal hyperplane that separates data points in a high-dimensional space. The LR model evaluates the statistical association between predictor variables and binary outcomes, providing interpretability for clinical decision-making. The XGBoost algorithm, based on gradient boosting, captures complex nonlinear relationships and is widely recognized for its strong performance in biomedical prediction tasks. RF, an ensemble learning method, reduces variance and enhances generalization by aggregating multiple decision trees. Finally, the KNN algorithm classifies samples according to the majority label among their k nearest neighbors in the feature space, offering simplicity and robustness for nonlinear datasets.

### 2.5 Model evaluation metrics

To comprehensively evaluate and compare the predictive performance of all models, multiple quantitative metrics were employed, including accuracy, sensitivity (recall), specificity, precision, F1-score, and the area under the receiver operating characteristic curve (AUC). Each metric provides complementary insight into classification performance: accuracy represents overall correctness; recall measures the proportion of correctly identified positive cases; precision quantifies the reliability of positive predictions; the F1-score balances precision and recall; and AUC reflects the model's overall discriminative ability across varying decision thresholds.

Hyperparameter tuning for each model was conducted using an exhaustive grid search combined with ten-fold cross-validation within the training dataset to achieve the optimal bias–variance trade-off. Model robustness was further validated using the independent test set, and 1,000 bootstrap resampling iterations were performed to estimate 95% confidence intervals for all major performance indicators. This multi-metric and multi-stage validation framework ensures that the resulting models are not only statistically reliable but also clinically interpretable and stable across varying data conditions.

### 2.6 Statistic analysis

All statistical analyses were performed using R software (version 4.3.0, https://www.R-project.org) and Python (version 3.8.0, https://www.python.org). Continuous variables were standardized prior to modeling to ensure consistent scaling across all features. Patients with missing essential variables were excluded to maintain data integrity. Outliers exceeding three standard deviations from the mean were identified and reviewed; biologically implausible values were removed, whereas clinically justified extreme values were retained to preserve real-world variability. All preprocessing procedures—including missing-data screening, outlier management, and Z-score standardization—were performed before model fitting and applied uniformly across all algorithms.

For continuous variables, the Shapiro–Wilk test was applied to assess normality. Normally distributed data were analyzed using two-tailed Student's t-*t*ests, whereas non-normally distributed data were compared using Mann–Whitney *U*-tests. Categorical variables were compared using Chi-square or Fisher's exact tests, as appropriate. Statistical significance was defined as *P* < 0.05.

Hyperparameter optimization for all models was conducted through grid search with ten-fold cross-validation within the training set to ensure stability and prevent overfitting. Model performance variability was quantified using 1,000 bootstrap iterations on the independent test set to estimate 95% confidence intervals (CIs) for major metrics (AUC, accuracy, recall, precision, specificity, and F1-score). Comparative analyses of AUC values among the six models were performed using DeLong's test implemented in the pROC package (version 1.18.5) in R.

Model interpretability was further enhanced through the SHapley Additive exPlanations (SHAP) method, which quantified each variable's contribution to prediction outcomes. Positive and negative SHAP values indicate the direction and magnitude of each feature's impact on the model output. The R packages used for data analysis and visualization are summarized in Table 1.

**Table 1:** Detailed information about the packages used in machine learning models.

Package names	Version	Description
Caret	6.0–86	Provides a suite of tools to streamline the process of training and tuning machine learning models
ggplot2	3.3.3	An implementation of the Grammar of Graphics in R, useful for creating complex multi-plot layouts
dplyr	1.0.6	A grammar of data manipulation, providing a consistent set of verbs that help you solve the most common data manipulation challenges
lattice	0.20–41	A powerful and elegant high-level data visualization system, inspired by Trellis graphics, for R
kknn	1.3.1	Weighted k-Nearest Neighbors for classification, regression, and clustering
rpart	4.1–15	Recursive partitioning for classification, regression, and survival trees
pROC	1.18.0	Likely a placeholder or error as “PROC” is not a standard R package; could be referencing SAS PROC from another context
randomForest	4.6–14	Breiman and Cutler's Random Forests for Classification and Regression
rmda	1.6	Risk Model Decision Analysis. Tools for performing decision analysis with risk models
readxl	1.3.1	Read Excel files (.xlsx and.xls) into R without external dependencies
xgboost	1.3.3	Extreme Gradient Boosting, which is an efficient implementation of gradient boosting framework

### 2.7 Data balancing and feature optimization

A total of 371 patients were included in this study, comprising 55 cases with lung metastases and 316 cases without. Because positive cases accounted for only 14.8% of the dataset, the severe class imbalance could have reduced model generalization and increased the risk of overfitting if not properly addressed. To mitigate this issue, the Synthetic Minority Over-sampling Technique (SMOTE) was applied to the training dataset using the smotefamily package (version 1.3.1) in R (version 4.3.0), following the method proposed by Chawla et al. (32). SMOTE generates synthetic minority samples by interpolating between existing ones, thereby increasing the representation of the minority class while maintaining the underlying feature distribution. The borderline-SMOTE algorithm was specifically employed to enhance data balance around class boundaries, improving minority class learning and preventing model bias.

Importantly, SMOTE was applied only to the training set after the 70:30 data split, rather than to the entire dataset, to avoid data leakage. Oversampling before splitting could introduce synthetic patterns into the test set, artificially inflating model performance. This post-split resampling strategy ensured that model evaluation reflected true real-world predictive capability while maintaining the natural data distribution for validation.

Following data balancing, feature screening and optimization were performed before model construction. Multivariate logistic regression was first used to identify clinically relevant predictors of lung metastasis, and eleven variables were ultimately retained for model development. To ensure compatibility with each algorithm's intrinsic characteristics, model-specific feature optimization was embedded within the machine learning pipeline. For Logistic Regression, L2 regularization was incorporated during grid search to prevent overfitting and reduce the influence of weak predictors. For SVM and KNN, recursive feature elimination (RFE) was conducted within cross-validation to determine the optimal subset of features. For tree-based models, including Random Forest and XGBoost, intrinsic feature importance ranking was utilized to evaluate predictor contributions. This integrated approach ensured that feature selection was tailored to each model, thereby enhancing interpretability, robustness, and overall generalizability of the predictive framework.

## 3 Results

### 3.1 Patient characteristics

A total of 371 patients were available in this study, the age ranges from 30 to 80 years old. The mean age of thyroid patients without lung metastases was 49 (48.91 ± 15.78). The average age of thyroid patients with lung metastases was 50 (49.57 ± 11.28). The total number of male patients is 137, accounting for 36.9% of the total number of thyroid patients. The total number of female patients was 234, accounting for 63.1% of the total number of patients. Among the thyroid patients with lung metastases, 26 cases (47.3%) were males and 29 cases (52.7%) were females, while for thyroid patients without metastases, 111 cases (35.1%) were males and 205 cases (64.9%) were females.

N stage is one of the most important pathological characteristics of the patients with thyroid cancer. The thyroid patients with lung metastases in T1 stage is 13, accounting (23.6%), the number for T2, T3, T4 followed by were 17 (30.9%), 5 (9.1%), and 20 (36.4%) respectively. While for thyroid patients without lung metastases in T1, T2, T3 and T4, the numbers were 218 (69.0%), 56 (17.7%), 5(1.6%) and 37 (11.7%) respectively.The detailed information was shown in Table 2.

**Table 2:** The detailed demographic information of the patients with thyroid cancer.

Categories	Lung metastasis	None Lung metastasis	*P* value
n	55 (14.8%)	316 (85.2%)	
Sex			
Male	26 (47.3%)	111 (35.1%)	<0.001
Female	29 (52.7%)	205 (64.9%)	
Age			
30–55	33 (60%)	209 (66.1%)	<0.001
55–80	22 (40%)	105 (33.9%)	
T stage			
T1	13 (23.6%)	218 (69.0%)	<0.001
T2	17 (30.9%)	56 (17.7%)	
T3	5 (9.1%)	5 (1.6%)	
T4	20 (36.4%)	37 (11.7%)	
N stage			
0	5 (9.1%)	0	<0.001
1	50 (89.9%)	316 (100%)	

**Figure 2:**
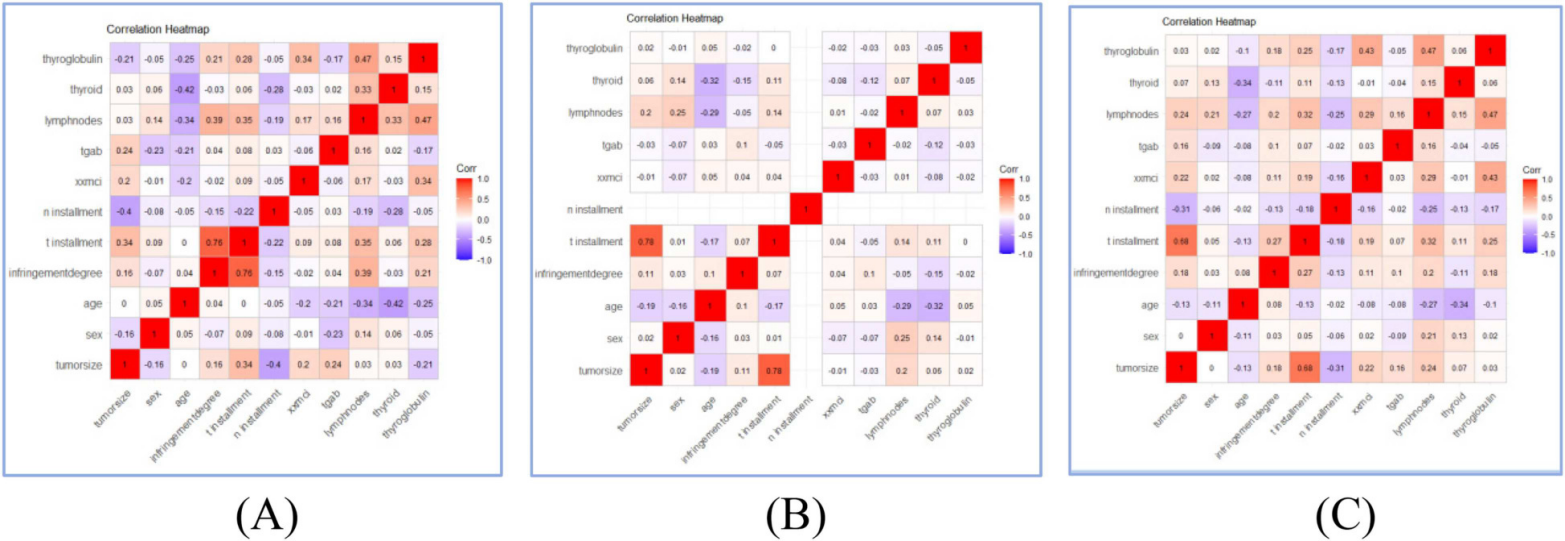
Correlation heatmaps of patients’ characteristics features in experimental group **(A)**, control group **(B)**, and mix group **(C)**.

11 variables were selected in the analysis, including sex, age, N stage, T stage, lymph node metastases count, tumor size, TgAb, degree of invasion, Thyroid Stimulating Hormone, thyroglobulin, Activity. The correlation between these variables were shown in Figure 2. The three correlation heatmaps were conducted based on experimental group, control group and the mix group respectively. In the heatmap related to the control group, the correlation coefficients for the N stage are not displayed. The primary reason is that the N stage values for the control group are all zero, hence no correlation coefficient exists. However, this does not affect the overall presentation of the correlations between variables. The result showed that all the variables were significantly different between the two groups (all *P* < 0.001), detailed information were shown in Table 2. Univariate logistic analysis and Multivariate logistic regression showed that all these variables were independently related with Occult pulmonary metastases (Tables 3, 4).

**Table 3:** Univariate analysis of clinical and biochemical factors associated with occult lung metastasis in DTC patients.

Factor	OR	95% CI	*P* Value
sex	1.276	[1.14, 1.68]	<0.001
age	1.46	[1.51, 1.83]	<0.001
Tumor size	1.976	[1.49, 2.60]	<0.001
degree of invasion	1.781	[1.42, 2.45]	<0.001
T stage			
T 1	0.682	[0.14, 3.31]	0.06
T 2	5.9	[6.13, 13.11]	<0.001
T 3	37	[20.87, 66.21]	<0.001
T 4	552	[231.30, 684.80]	<0.001
N stage			
N0	Reference		
N1	7.320	[7.32, 9.47]	<0.001
administrated activity	2.803	[1.90, 4.20]	<0.001
TgAb	1.427	[1.06, 1.90]	<0.001
lymph node metastases count	3.257	[2.30, 4.59]	<0.001
TSH	1.07	[1.05, 1.21]	<0.001
Tg	4.829	[2.61, 8.90]	<0.001

Univariate analysis of clinical and biochemical variables evaluating their association with the presence of occult lung metastasis in DTC patients. *P* values indicate the statistical significance of group differences.

Tg, thyroglobulin; TSH, thyroid-stimulating hormone; RF, Random Forest; DTC, differentiated thyroid cancer.

**Table 4:** Multivariate analysis of variables related to lung metastasis. .

Factor	OR	95% CI	*P* Value
Gender	1.603	[1.54, 3.1]	<0.001
Age	1.046	[1.01, 1.08]	<0.001
Tumor size	1.291	[1.15, 2.20]	<0.001
degree of invasion	1.781	[1.05, 3.15]	<0.001
T stage			
T 1	0.682	[0.14, 3.31]	0.635
T 2	4.2	[5.82, 10.06]	<0.001
T 3	34	[18.71, 61.50]	<0.001
T 4	456	[187.10, 615.12]	<0.001
N stage			
N0	Reference		
N1	7.320	[7.32, 9.47]	<0.001
Administrated activity	1.41	[1.26, 1.07]	<0.001
TgAb	1.12	[1.06, 2.13]	<0.001
lymph node metastases count	1.113	[1.05, 1.15]	<0.001
TSH	1.17	[1.09, 2.12]	<0.001
Tg	1.025	[1.01, 1.03]	<0.001

Multivariate logistic regression identifying independent predictors associated with occult lung metastasis in DTC patients. Variables with *P* < 0.05 in univariate analysis were included.

OR, odds ratio; CI, confidence interval; Tg, thyroglobulin; TSH, thyroid-stimulating hormone; DTC, differentiated thyroid cancer.

Multivariate logistic regression analysis (Table 4) identified several independent predictors significantly associated with occult lung metastasis in patients with differentiated thyroid carcinoma (DTC). Among these variables, lymph node metastases count (OR = 1.113, 95% CI: 1.05–1.15, *P* < 0.001) and thyroglobulin (Tg, OR = 1.025, 95% CI: 1.01–1.03, *P* < 0.001) exhibited the strongest associations with metastasis risk. These findings suggest that an increasing number of metastatic lymph nodes and elevated Tg levels substantially raise the likelihood of undetected lung metastases before 131I therapy. Clinically, this reflects the dual influence of anatomical spread (via lymphatic dissemination) and functional tumor activity (through iodine-avid Tg-producing cells) on disease progression.

In addition, advanced T and N stages, larger tumor size, and higher degree of invasion were also significantly correlated with the occurrence of occult metastasis, highlighting that both local tumor aggressiveness and systemic dissemination contribute to the metastatic phenotype. Elevated TSH levels further amplified this risk, consistent with its known role in stimulating thyroid follicular cell proliferation and promoting iodine uptake.

These multivariate findings are in line with the SHAP feature importance results derived from the logistic regression model, in which lymph node metastases count and Tg ranked as the most influential predictors. Together, the regression and SHAP analyses provide complementary evidence that both structural (tumor invasion and nodal spread) and biochemical (Tg and TSH activity) markers jointly define the metastatic potential of DTC. This convergence between classical statistical analysis and explainable machine learning reinforces the robustness and biological plausibility of the predictive framework established in this study.

### 3.2 Model performance

**Figure 3:**
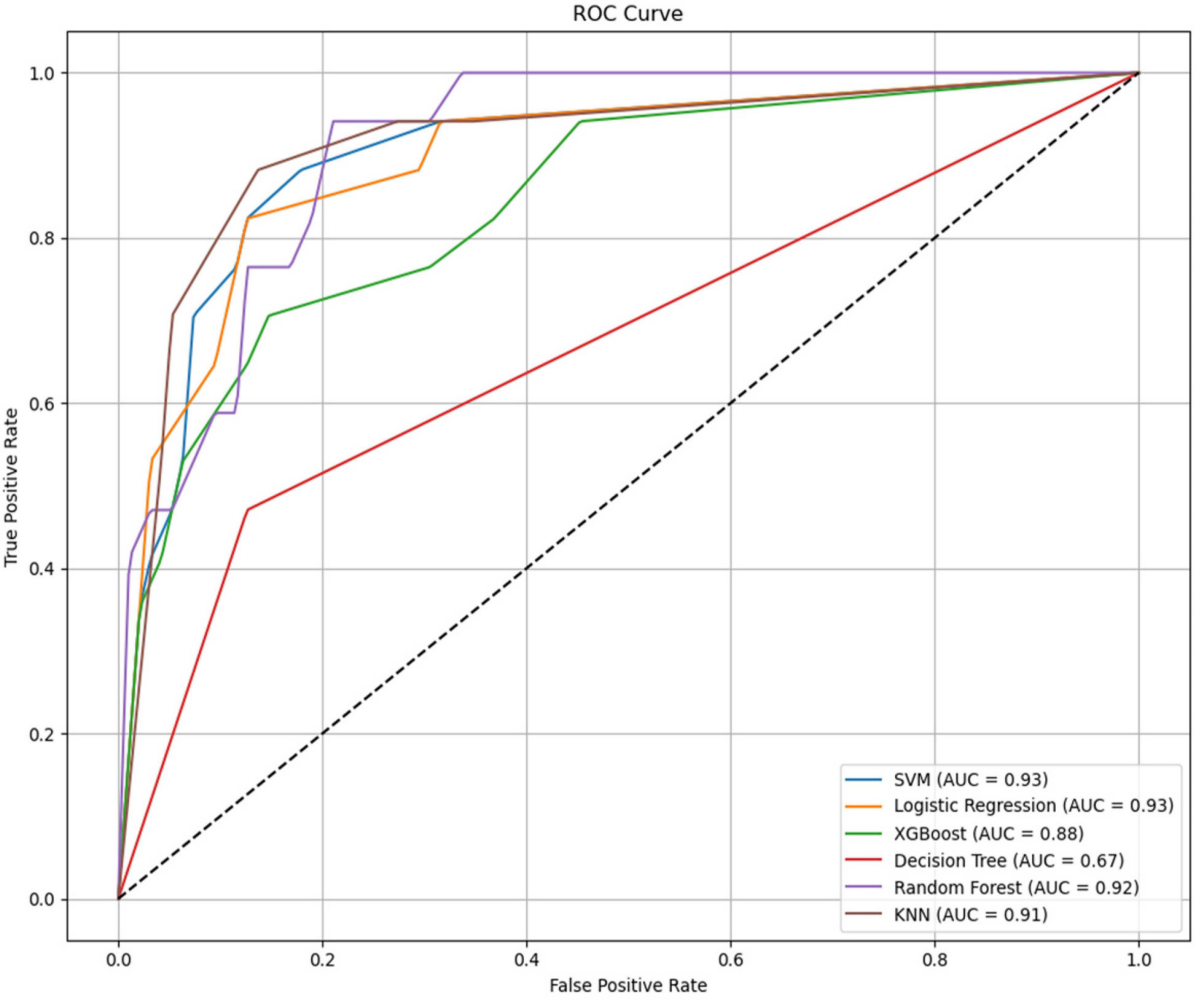
Receiver operating characteristic (ROC) curves comparing the classification performance of six machine learning models—logistic regression (LR), support vector machine (SVM), random forest (RF), k-nearest neighbors (KNN), XGBoost, and decision tree (DT)—for predicting occult lung metastasis in differentiated thyroid cancer (DTC) patients. The area under the curve (AUC) values for each model are displayed in the legend, illustrating overall discriminative ability. AUC, area under the curve; LR, logistic regression; SVM, support vector machine; RF, random forest; KNN, k-nearest neighbors; DT, decision tree; DTC, differentiated thyroid cancer.

Gender,Age, Tumor size, Degree of invasion, T stage, N stage, Administrated activity, TgAb, lymph node metastases count, TSH, Tg were conducted in the model. We used the SMOTE method to equilibrate the data set before modeling. Six machine learning models were developed and compared based on learning, receiver operating characteristic (ROC), the result was shown in Figure 3. It is easy the see that SVM had the highest AUC value of 0.93, LR had an AUC value of 0.93; RF had an AUC value of 0.92; KNN had a AUC value of 0.91, XGBoost had a AUC value of 0.87; Decision Tree had a AUC value of 0.67. Statistical comparison of AUC values using DeLong's test revealed no significant difference (*P* > 0.05) among the top-performing models—Logistic Regression, SVM, and KNN—indicating that their discriminative abilities were statistically comparable.

However, AUC was not the only indicator for evaluating model performance, The clinical applicability of all models was further assessed using Sensitivity, specificity, precision, accuracy and F1 Score. The result was shown in Table 5. All reported metrics are presented as mean values with corresponding 95% confidence intervals, providing an estimate of model stability and reliability across bootstrap iterations. The six machine learning models exhibited balanced accuracy ranging from 0.81 to 0.92. Among them, the logistic regression (LR) model achieved the highest precision (0.93) and specificity (0.97), indicating strong reliability when identifying high-risk cases, whereas the random forest (RF) model showed the highest recall (0.94), demonstrating strong sensitivity in detecting metastatic cases. Each model demonstrated strong performance across different evaluation metrics. RF achieves the highest sensitivity (so called recall), LR had the highest specificity, precision, accuracy and F1 Score. As SVM model and LR model has similar AUC values, this result showed that LR algorithm model has the most powerful function.

**Table 5:** Performance of various prediction models predicting lung metastasis thyroid cancer using a testing data set (All values are reported to two decimal places).

Models	Sensitivity (Recall)	Specificity	Precision	Accuracy	F1 Score	AUC
SVM	0.76 [0.60–0.88]	0.92 [0.86–0.96]	0.65 [0.48–0.79]	0.90 [0.84–0.95]	0.70 [0.56–0.81]	0.93 [0.88–0.97]
LR	0.65 [0.48–0.79]	0.96 [0.91–0.99]	0.92 [0.86–0.97]	0.91 [0.85–0.95]	0.70 [0.57–0.80]	0.93 [0.88–0.97]
XGBoost	0.58 [0.41–0.74]	0.93 [0.87–0.97]	0.62 [0.46–0.77]	0.88 [0.82–0.93]	0.60 [0.48–0.70]	0.87 [0.82–0.92]
DT	0.47 [0.31–0.64]	0.87 [0.79–0.92]	0.40 [0.26–0.56]	0.81 [0.74–0.87]	0.43 [0.33–0.54]	0.67 [0.57–0.77]
RF	0.94 [0.83–0.99]	0.81 [0.72–0.88]	0.47 [0.34–0.62]	0.83 [0.77–0.89]	0.62 [0.51–0.72]	0.92 [0.86–0.96]
KNN	0.70 [0.54–0.83]	0.94 [0.88–0.98]	0.70 [0.53–0.84]	0.91 [0.85–0.95]	0.70 [0.59–0.80]	0.91 [0.86–0.94]

Comparison of six machine learning models—Logistic Regression (LR), Support Vector Machine (SVM), Random Forest (RF), k-Nearest Neighbors (KNN), XGBoost, and Decision Tree (DT)—for predicting occult lung metastasis. Reported metrics include accuracy, precision, recall, specificity, F1-score, and AUC. All values are expressed with two decimal places to reflect data precision.

LR, logistic regression; SVM, support vector machine; RF, random forest; KNN, k-nearest neighbors; DT, decision tree; AUC, area under the curve; F1, F1 score.

### 3.3 Model interpretability

**Figure 4:**
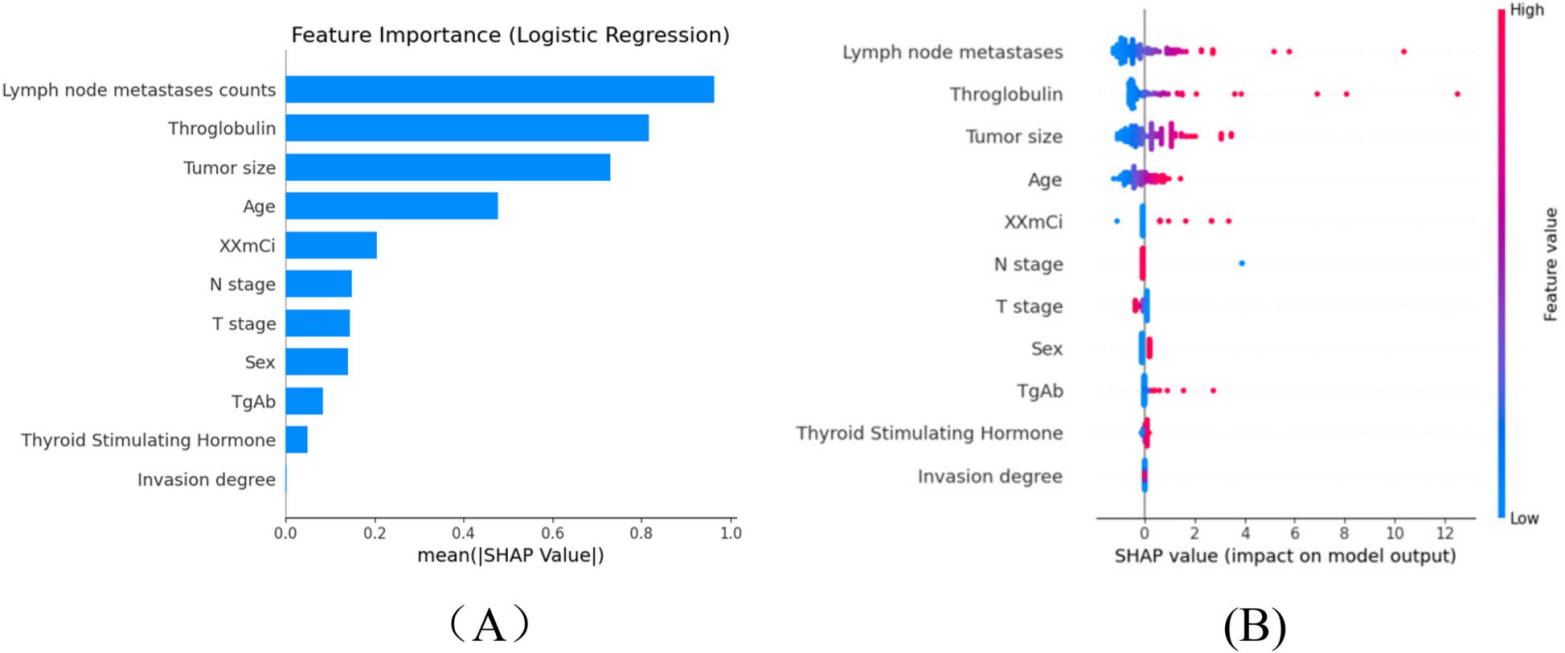
The shapley additive exPlanations values of the better prediction model, LR. **(A)** Average impact of features on model. **(B)** Detailed impact analysis of each feature predictions. Each dot represents a single patient, with color indicating feature value (red = high, blue = low). Lymph node metastasis count, thyroglobulin (Tg), and thyroid-stimulating hormone (TSH) emerged as the most influential predictors of occult lung metastasis, reflecting their known clinical significance in thyroid cancer progression. SHAP, SHapley additive exPlanations; Tg, thyroglobulin; TSH, thyroid-stimulating hormone; RF, random forest; SVM, support vector machine; LR, logistic regression; DTC, differentiated thyroid cancer.

Machine learning models are often considered “black boxes”, which makes their internal decision-making processes difficult to interpret. To enhance the model's interpretability, SHAP analysis were conducted and explanations were provided: global explanations at the features level. As shown in the SHAP summary plot (Figure 4), features were evaluated for their contributions to the model using average SHAP values, displayed in descending order, which displayed the positive and negative impact of each feature. Figure 4 (left) displayed the absolute values of the average SHAP values for different features. lymph node metastases count had the most significant impact on model output, followed by throglobulin,tumor size, age, administrated activity, N stage, T stage, sex, Tgab, thyroid stimulating, invasion degree. Figure 4 (Right) provides a more detailed view of the impact of each feature on individual predictions.

To enhance the interpretability of the SHAP analysis, we have now provided detailed SHAP summary and dependence plots (new Figures 4A–C) to visualize both global and local feature impacts. The SHAP summary plot ranks the eleven predictors by their mean absolute SHAP values, while the dependence plots illustrate how variations in thyroglobulin (Tg), lymph node metastases count, and TSH influence the prediction output. As shown in the updated figure, higher Tg and lymph node counts markedly increase the predicted probability of occult lung metastasis, confirming that patients with elevated biochemical tumor load and greater nodal involvement are at higher metastatic risk.

From a biological and clinical perspective, these findings are consistent with known mechanisms of differentiated thyroid carcinoma (DTC) dissemination. Lymph node metastases represent the first step in extra-thyroidal spread, reflecting local invasion and lymphatic drainage disruption, which often precedes hematogenous lung metastasis. Elevated serum Tg reflects residual or metastatic thyroid tissue with preserved iodine-avid function and is a sensitive biochemical marker of occult disease, even when imaging is negative. Elevated TSH further promotes tumor cell proliferation and iodine uptake through upregulation of the sodium-iodide symporter, which may potentiate the progression of micrometastatic lesions. Together, these features capture both the anatomical (nodal spread) and functional (biochemical activity) pathways of metastatic progression, explaining their dominant contribution to model prediction.

By integrating these biological insights with SHAP-based interpretability, our model bridges statistical feature attribution and clinical pathophysiology, allowing physicians to understand why specific features drive metastasis prediction. This interpretive transparency enhances the model's clinical trustworthiness and supports its use as a decision-support tool for early identification of high-risk patients before 131I therapy.

**Figure 5:**
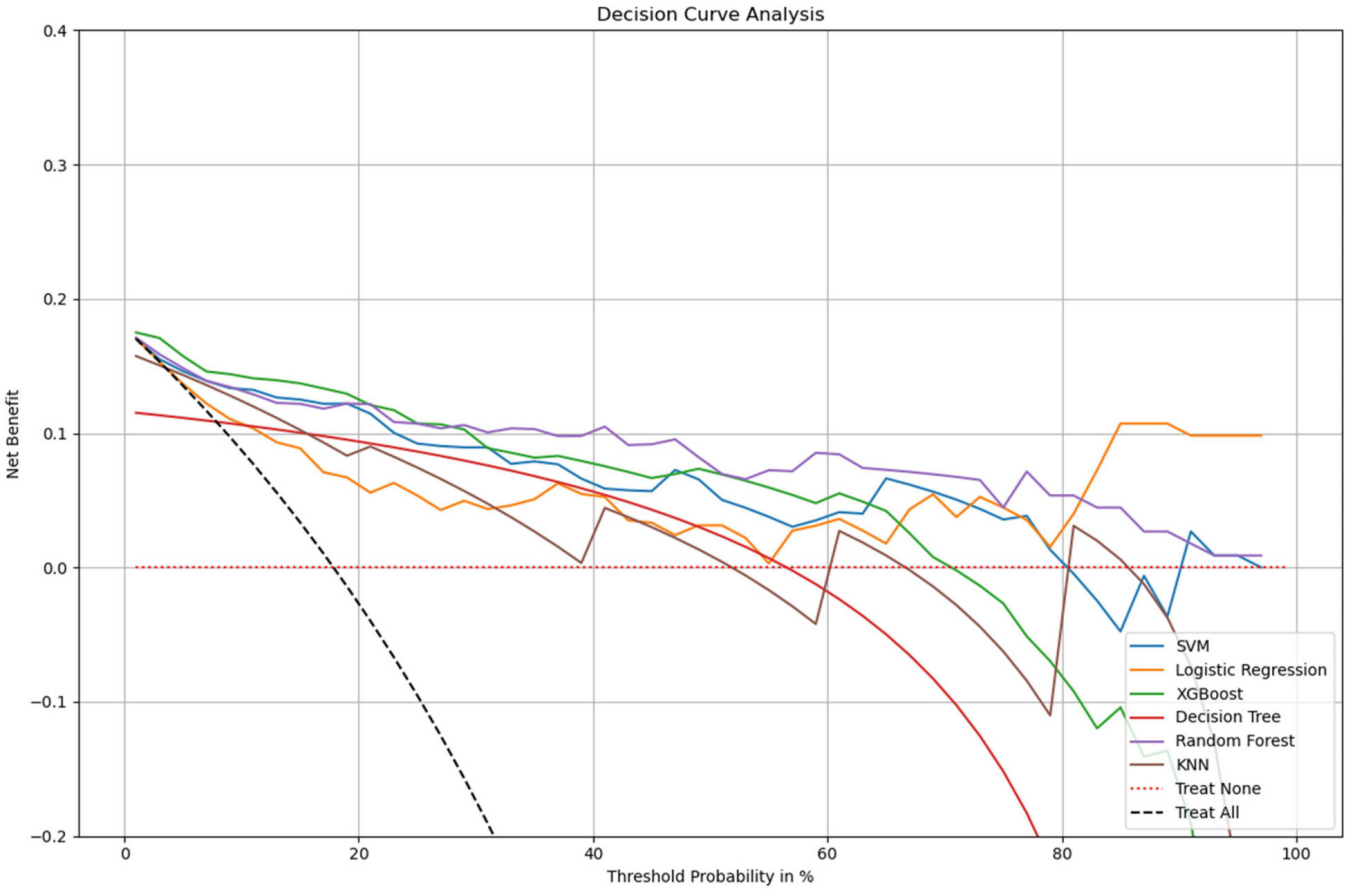
Decision curve analysis for multiple models. *x*-axis: the threshold probabilities, which indicate the point at which the predicted risk of metastasis leads to intervention. *y*-axis: measures the net benefit, calculated by weighing the true positives against the false positives, where the latter are penalized more heavily at lower threshold probabilities. Treat All: the net benefit if all patients were treated, assuming every patient has the condition. Treat None: the net benefit if no patients were treated, assuming no patients have the condition.The logistic regression (LR) model demonstrates the highest overall net benefit within the clinically relevant threshold range (0.2–0.8), indicating strong practical utility for decision support in predicting occult lung metastasis. DCA, decision curve analysis; LR, logistic regression; RF, random forest; SVM, support vector machine; DT, decision tree; KNN, k-nearest neighbors; AUC, area under the curve.

This study further performed a decision curve analysis (DCA). Net benefit (NB) is the percentage of the net positives in the total sample. There are two special lines, namely Treat all and Treat none, are used as reference lines. The model only holds practical value at a specific threshold probability if its Net Benefit (NB) exceeds that of both the Treat All and Treat None strategies. As shown in Figure 5, we can conclude that all models had higher net returns than the two extreme lines in the 0 to 1 threshold range. we observe that Logistic Regression demonstrates consistently higher net benefit across a wide range of threshold probabilities compared to other models, suggesting it is the most effective at maximizing the benefits of true positive results while minimizing the harm of false positives. Most models begin to decline in net benefit as the threshold probability increases, which is typical, as higher thresholds require higher certainty in predictions, reducing the number of true positives identified. Logistic Regression may offer the most pragmatic balance for clinical application by providing substantial net benefit without incurring the risks associated with over-treatment or under-treatment across a practical range of clinical decision thresholds. This analysis emphasizes the need to align model selection with clinical priorities, balancing early detection benefits against potential overtreatment risks.

## 4 Discussion

In this study, correlation heatmaps were employed to explore inter-variable relationships within both the experimental and control groups. While most features exhibited relatively low to moderate correlations, some pairs—such as tumor size and T stage, or Tg and TSH—showed notable collinearity. Although these relationships reflect expected clinical dependencies, they also raise concerns about potential multicollinearity in the predictive modeling process. Given the relatively small dataset size, all performance metrics were reported to two decimal places to better reflect the practical level of precision supported by the data. Minor numerical fluctuations are expected across resampling iterations, and the rounded values represent robust central tendencies rather than exact point estimate.

Multicollinearity can inflate the variance of coefficient estimates in models like logistic regression, reduce the interpretability of individual feature contributions, and increase the risk of overfitting. In our analysis, this risk was partially mitigated by using machine learning models that are less sensitive to multicollinearity, such as Random Forest and XGBoost. Nonetheless, the logistic regression model, which forms the basis of our primary interpretation, may still be affected to some extent.

In this study, eleven acceptable features were selected by multivariate analysis and six machine learning models consisting of SVM, LR, XGBoost, DT, RF and KNN were designed to predict the lung metastasis in patients with differentiated thyroid cancer (DTC) based on the clinical data from Zhejiang cancer hospital. We also used SMOTE method to make the dataset balanced and then utilized comprehensive scoring indicators, such as sensitivity, accuracy, precision, specificity, F1 Score and AUC score to improve model performance. The result showed that among the six machine learning models, SVM and LR models showed the highest AUC (0.93) and wonderful clinical applicability. The F1 values and accuracy of LR, KNN, and SVM models are ranked in the top three, the values were (0.70, 0.70, 0.70) and (0.91, 0.91,0.90) respectively. LR models had the highest precision value which approximation of 0.94, followed by KNN model with the value of 0.70 and SVM with the value of 0.65, unfortunately, except for XGBoost model had a precision value of 0.62, the other three models showed worse performance with the values even lower than 0.5. The LR, KNN and XGBoost model ranked the top three in specificity, the values were 0.96, 0.94 and 0.93 respectively. However, RF model won the highest recall values of 0.94, SVM and KNN models followed. Hence, we believe that accuracy can not be regard as the only scoring indicator for model performance evaluation in unbalanced classification problems.

In the context of class imbalance, relying solely on AUC may obscure important differences in model performance for minority classes. Although the Logistic Regression (LR) model achieved a high AUC of 0.93 it exhibited a moderate sensitivity (recall) of 0.64 compared to the Random Forest (RF) model, which demonstrated the highest recall of 0.94 but lower precision. Precision, which reflects the proportion of true positives among predicted positives, was highest in the LR model (0.92), indicating strong reliability when the model predicts lung metastasis. Meanwhile, the F1-score—a harmonic mean of precision and recall—was comparable between LR, SVM, and KNN models, highlighting the trade-off between sensitivity and precision across models. The superior performance of logistic regression, despite its simplicity, reflects the linear and well-structured nature of the selected clinical predictors. In datasets with moderate feature dimensions and clear variable associations, simpler models may achieve comparable or even higher generalizability compared with complex ensemble algorithms.

Given these results, model selection should not be based solely on AUC but rather on a holistic view of all evaluation metrics. The LR model was ultimately preferred not only for its high AUC and specificity but also for its clinical interpretability and practical decision-support potential, as confirmed by its superior performance in the Decision Curve Analysis (DCA). However, in scenarios where maximizing sensitivity is paramount (e.g., to avoid missing lung metastases), RF or SVM models might be more appropriate, albeit at the cost of lower precision or specificity. This nuanced evaluation underscores the importance of aligning model choice with clinical priorities and the cost of false positives or negatives. Although the logistic regression model achieved high overall accuracy and specificity, its recall value indicates that a proportion of occult metastases may remain undetected. This sensitivity–specificity trade-off reflects a deliberate design choice to favor clinical reliability and interpretability over maximal sensitivity. Future work will explore hybrid or ensemble frameworks that combine the interpretability of LR with the higher sensitivity of tree-based models to improve overall clinical utility. It is also important to acknowledge that the moderate recall (0.64) of the logistic regression model indicates a potential limitation in identifying all true metastasis cases. From a clinical perspective, this means that while the model provides high confidence in positive predictions (high precision), some patients with occult metastases may still be missed, particularly when lesions are small or biochemically silent. This trade-off reflects the inherent balance between false-positive reduction and sensitivity improvement in diagnostic models. In future studies, additional optimization—such as adjusting classification thresholds, incorporating cost-sensitive learning, or combining logistic regression with more sensitive ensemble methods—will be explored to enhance case detection while maintaining interpretability.

Furthermore, SHAP analysis was conducted to evaluate the relative importance of individual features in the predictive models. The results revealed that lymph node metastasis count and thyroglobulin (Tg) were the most influential variables contributing to the prediction of 131I-WBS outcomes. These findings have strong pathophysiological underpinnings and reinforce the translational value of the model. The interpretability framework established by SHAP facilitates the conversion of machine learning outputs into clinically actionable insights, thereby enhancing the model's reliability and transparency in real-world thyroid cancer management.

The lymph node metastasis count serves as a direct indicator of tumor burden and aggressiveness. As part of the lymphatic system, lymph nodes are among the earliest and most frequent sites of metastatic spread (22). The presence of nodal metastases signifies a breach in local tumor containment and often precedes distant dissemination, including to the lungs (23, 24). In clinical practice, a higher lymph node metastasis count is strongly correlated with a greater probability of distant metastases detectable by 131I-WBS, which is sensitive to iodine-avid thyroid cancer cells (25). Thus, this variable captures the anatomical dimension of metastatic risk and is consistent with established clinical observations.

Thyroglobulin (Tg), a glycoprotein secreted exclusively by thyroid follicular cells, is a key biomarker for postoperative surveillance and recurrence monitoring in differentiated thyroid carcinoma (DTC) (26, 27). Elevated Tg levels serve as an early indicator of residual or metastatic thyroid tissue that remains metabolically active but may not yet be radiologically detectable (28–30). Because Tg directly reflects thyroid cell activity, its elevated post-treatment levels signify biologically active, iodine-avid tumor remnants likely to result in positive 131I-WBS findings. The strong SHAP contribution of Tg in our model therefore aligns with its established role as a biochemical surrogate for disease persistence and metastatic potential.

In addition to Tg and lymph node involvement, thyroid-stimulating hormone (TSH) emerged as another variable with substantial predictive importance. TSH plays a central role in stimulating thyroid follicular cell proliferation and enhancing iodine uptake via the sodium/iodide symporter pathway. Elevated TSH levels have been associated with increased recurrence and distant metastasis risks in DTC (15, 18), while strict TSH suppression therapy has been shown to mitigate such risks (31). Hence, the high SHAP value of TSH underscores not only its predictive significance but also its biological and therapeutic relevance in thyroid cancer progression.

The consistency between SHAP-derived importance and established clinical mechanisms strengthens the interpretability and credibility of the model. Elevated Tg and TSH jointly suggest the presence of metabolically active thyroid remnants or micrometastatic lesions, while a high lymph node burden indicates structural dissemination potential. Together, these variables encapsulate both functional and anatomical determinants of metastasis, confirming that the model captures biologically meaningful and clinically relevant features of occult metastatic disease. Similar interpretations have been reported in prior studies integrating molecular and serological predictors for metastatic thyroid carcinoma (5, 7, 18).

Additionally, the SHAP plot revealed that the variable sex exhibited a binary influence pattern, suggesting distinct prediction distributions between male and female patients. Although sex-specific hormonal and physiological factors may influence metastatic behavior, the limited number of male cases precluded constructing separate sex-stratified models in this study. A unified model was therefore adopted to maintain statistical robustness and model comparability. Future multicenter studies with larger, balanced cohorts could explore sex-stratified modeling to evaluate potential gender-related heterogeneity in metastatic risk.

Finally, while all eleven features were retained to ensure comparability across machine learning algorithms and to capture potential nonlinear interactions, we acknowledge that excluding consistently low-impact variables could streamline the model and enhance computational efficiency. Future optimization efforts will evaluate feature reduction strategies to balance simplicity, interpretability, and predictive performance.

Overall, these results underscore that the most influential predictors—lymph node metastasis count, Tg, and TSH—represent clinically and biologically meaningful indicators of metastatic potential. Their prominence within the model validates the underlying pathophysiological mechanisms and highlights the capacity of the logistic regression framework to yield interpretable, clinically relevant predictions that can inform individualized management strategies in patients with differentiated thyroid cancer.

### 4.1 Limitation and future improvement

This study conducted at Zhejiang Cancer Hospital provided important insights into predicting ^131^I-WBS outcomes using logistic regression models based on significant predictors such as lymph node metastases count counts and thyroglobulin levels. However, there are several limitations that need to be addressed for future research to enhance the model's applicability and accuracy.

Firstly, the study's predictive model is derived from a single institution's patient population, which may limit the generalizability of the findings. Patient demographics, treatment protocols, and diagnostic practices can vary significantly across different regions and institutions, potentially affecting the model's performance in external populations. Future studies should consider validating the model across multiple centers to confirm its effectiveness and reliability in diverse clinical settings. Given the limited subgroup sample sizes in certain variables (e.g., *n* = 5), the calculated *P*-values should be interpreted with caution, as small-sample testing may yield unstable estimates of significance. Therefore, these statistical comparisons are provided mainly to illustrate feature distributions rather than to draw inferential conclusions.

Secondly, the dataset was collected over an extended period, which may introduce minor temporal variations due to gradual improvements in imaging and laboratory technologies. To minimize this potential bias, all patients were diagnosed and treated within the same institution under standardized diagnostic and ¹³¹I treatment protocols. The same imaging modality (131I-WBS) and laboratory assay methods for thyroglobulin and TSH were consistently applied and validated by the hospital's central laboratory. Moreover, as the model utilized normalized clinical and biochemical parameters rather than raw imaging data, the influence of technological upgrades on model performance is expected to be minimal. Nevertheless, future multicenter and prospective studies are warranted to evaluate the model's robustness across different technological settings and evolving diagnostic standards.

Thirdly, the model relies primarily on specific clinical markers that, while informative, do not encompass all potential determinants influencing 131I-WBS outcomes. For instance, genetic or molecular biomarkers that play essential roles in thyroid cancer progression were not included in this study. Incorporating these variables may provide a more comprehensive representation of metastatic risk.

Furthermore, the logistic regression framework, though interpretable and robust, may not fully capture complex nonlinear interactions among predictors compared with advanced ensemble or deep learning algorithms. Future research could explore hybrid models that combine interpretability with the flexibility of nonlinear learners to improve predictive accuracy.

The retrospective nature of this study also introduces potential biases related to data completeness and patient selection. Prospective validation in real-time clinical workflows will be essential to confirm the model's predictive reliability and clinical utility. Additionally, although stimulated thyroglobulin (sTg) is a more sensitive indicator for persistent disease, inconsistent stimulation protocols limited its inclusion, and only baseline Tg values were analyzed. Future studies should standardize sTg measurements to improve subgroup stratification.

In this study, SHAP analysis was employed to enhance the interpretability of the logistic regression model by identifying and ranking feature contributions. This approach confirmed that critical predictors such as lymph node metastasis count, Tg, and TSH were consistent with known biological mechanisms of thyroid cancer metastasis.

However, we acknowledge that SHAP analysis was not performed for the more complex models used in this study, such as Random Forest (RF), Support Vector Machine (SVM), and XGBoost. These models—despite demonstrating strong predictive performance—are often regarded as “black-box” methods due to their lack of inherent interpretability. The absence of comparable explanation techniques for these models limits our ability to fully compare model behavior and may hinder clinical acceptance. Although SMOTE was employed to alleviate class imbalance and enhance model generalizability, it cannot fully substitute for real-world sample diversity. Future research should validate these findings using cost-sensitive learning or weighted loss approaches on larger, multicenter datasets.

Future work will focus on extending interpretability frameworks to these models. In particular, tree-based SHAP can be directly applied to RF and XGBoost, while kernel-based SHAP can be adapted for SVM. Providing feature-attribution explanations across all high-performing models will enhance the transparency, clinical utility, and decision-support trustworthiness of our predictive framework. Also future work will focus on multicenter external validation to confirm the reproducibility and robustness of the predictive model across different clinical settings and patient populations.

Moreover, we acknowledge that the current study used multivariate statistical analysis as an initial screening step for feature selection, which may not fully capture nonlinear relationships or interaction effects among predictors. Future work will focus on integrating model-embedded feature selection strategies—such as recursive feature elimination (RFE), L1/L2 regularization, and feature importance ranking—within the machine learning pipeline to achieve more objective and data-driven optimization of predictors.

Future studies may benefit from integrating molecular biomarkers (e.g., BRAF, TERT mutations) and imaging-derived features, including radiomics from CT or SPECT/CT data. Such multi-omics and imaging fusion approaches could capture tumor heterogeneity at both biological and structural levels, potentially improving early detection of occult metastases beyond what clinical variables alone can offer.

Addressing these limitations in future research could significantly enhance the predictive model's accuracy and clinical utility, ultimately aiding in the personalized management of thyroid cancer patients and improving their treatment outcomes.

## 5 Conclusion

This study demonstrated that machine learning models—particularly logistic regression (LR)—showed favorable overall performance in predicting occult lung metastasis among patients with differentiated thyroid cancer (DTC). The LR model achieved the highest AUC, specificity, and precision, indicating strong reliability when identifying high-risk cases. However, its moderate recall (0.6471) suggests that some true metastasis cases may remain undetected, highlighting the need for further optimization before clinical implementation. Among the variables included, thyroglobulin (Tg) and lymph node metastasis count were identified as the most influential predictors, consistent with their established roles in thyroid cancer progression. Future studies should focus on improving the model's sensitivity through techniques such as threshold adjustment, cost-sensitive learning, or hybrid ensemble approaches, while maintaining interpretability for practical clinical decision support.

## Data Availability

The dataset and meterials generated during the current study are available from the corresponding author on reasonable request.
